# Graphene Nanoplatelets as a Replacement for Carbon Black in Rubber Compounds

**DOI:** 10.3390/polym14061204

**Published:** 2022-03-17

**Authors:** James R. Innes, Robert J. Young, Dimitrios G. Papageorgiou

**Affiliations:** 1 Polymer IRC, Faculty of Engineering and Informatics, University of Bradford, Richmond Road, Bradford BD7 1DP, UK; j.innes1@bradford.ac.uk; 2 National Graphene Institute, Henry Royce Institute and Department of Materials, University of Manchester, Oxford Road, Manchester M13 9PL, UK; robert.young@manchester.ac.uk; 3 School of Engineering and Materials Science, Queen Mary University of London, London E1 4NS, UK

**Keywords:** nitrile butadiene rubber, graphene nanoplatelets, micromechanics, carbon black

## Abstract

In this work, we evaluated the processing and reinforcement characteristics of both carbon black (CB) and graphene nanoplatelets (GNPs) within a nitrile butadiene rubber (NBR) matrix. The aspect ratio of the GNPs was measured using atomic force microscopy (AFM) and related to the dispersion and agglomeration within the NBR matrix, as observed by scanning electron microscopy (SEM). The relationship between GNP aspect ratio and mechanical properties was studied by micromechanical modelling. The tensile and tear properties of NBR after compounding with GNPs were enhanced to a greater extent compared to carbon black, while curing times were smaller and scorch times longer, indicating some of the advantages of using GNPs. Overall, the inherent properties of GNPs along with their geometry led to the production of better-performing rubber compounds that can replace their CB-filled counterparts in applications where flexibility, tear strength and compliance are important. The influence of processing on dispersion, orientation and agglomeration of flakes was also highlighted with respect to the Young’s modulus of the NBR compounds.

## 1. Introduction

Within the oil and gas industry, there is a demand for chemical- and tear-resistant elastomer seals that can be easily fitted. Carbon black (CB) is commonly used as the reinforcing filler in nitrile chemically-resistant butadiene rubber (NBR) to achieve the desired tear resistance. However, this results in seals that are overly hard and inflexible, which in turn leads to installation damage during the fitting of NBR seals. The advantages of carbon black include its low cost, its ease of processing and its reinforcing effects on the produced materials, mostly originating from the strong interactions between the elastomer matrix and the filler [1]. These spherical CB particles are tens to hundreds of nanometres in size, depending on the selected CB grade. The activity of CB comes from surface groups, while its nanostructure may be thought of as onion-like with an amorphous core [2]. Carbon blacks have a number of oxygen-containing functional groups on their surface, which aid dispersion within a polymer matrix. These functional groups help increase the crosslink density and the subsequent properties of the elastomer, which is why carbon black is so widely used as a reinforcement in elastomeric materials.

The continuous demand for better-performing, multifunctional elastomer nanocomposites, along with the discovery of nanocarbons that are able to support these demands, such as multi-walled carbon nanotubes [3] and graphene [4], has led to a shift in research away from carbon black and onto new, high-performance fillers. The unique characteristics of graphene include its mechanical properties [5,6], its thermal/electrical conductivities [7,8] and its large surface area/aspect ratio [9], amongst others, making it attractive for use in a vast number of applications, including polymer nanocomposites. However, the industrial scale up of few-layer graphene and its high cost are currently restricting its widespread use. In this context, the majority of the published work and commercial applications use either graphene oxide (GO), reduced graphene oxide (rGO) or graphene nanoplatelets (GNPs) as reinforcements. GNPs especially have shown great promise as fillers in polymer nanocomposites since they perform similarly to few-layer graphenes [10,11,12,13,14,15] yet they are available at a reasonable price. In terms of mechanical reinforcement, as we pointed out in a previous paper, rather surprisingly, the modulus of the nanocomposite is independent of the modulus of the filler, while it scales with the modulus of the matrix [10]. The most significant parameters that can affect the final performance of the nanocomposites include aspect ratio and the orientation of the filler, along with the strength of the interface with the matrix.

Whilst some works have reported on the reinforcement of nitrile butadiene rubber (NBR) by graphene materials, the produced or procured graphene-related materials have not been sufficiently characterised in-situ and ex-situ in an attempt to model the micromechanics of the respective NBR composites. NBR was selected as it is commonly used within the oil and gas industry due to its resistance to oils and its relatively good mechanical properties [16]. Varghese et al. reinforced NBR with 1–5 phr GNPs by dry mixing and compared with low loadings of carbon black and hybrid combinations, showing improvements in scorch time, mechanical properties and gas barrier properties for GNPs compared with CB [17]. Various graphene oxides have also been used to reinforce NBR using solution-blending methods, leading to improved mechanical properties at low filler contents [18]. However, most authors have stuck to low loadings of GNPs ≤ 5 phr, which may not be suitable for demanding applications, and the micromechanical modelling of GNPs in NBR has not been fully evaluated [17,19,20,21,22,23,24].

In this work, we have reinforced a nitrile butadiene rubber (NBR) matrix with both graphene nanoplatelets of different diameters and carbon black. The novelty of this work arises from the initial ex-situ/in-situ measurement of the GNP aspect ratios and the use of these results within the corresponding micromechanics discussion. The aspect ratio of the GNPs was measured ex-situ by AFM and compared with the in-situ SEM results. Additionally, whilst many papers have attributed reduced mechanical properties at high graphene loadings to agglomeration, the reasoning had not been previously fully elaborated or demonstrated via micromechanical modelling and simultaneous measurement of the aspect ratios of GNPs. The relationship between change in the effective aspect ratio due to agglomeration and filler loading is presented. For this purpose, the tensile, tear and hardness properties were thoroughly evaluated and micromechanical models were applied to the modulus results. This work aims to evaluate the mechanical behaviour for each set of samples reinforced with different size GNPs, as well as CB, and elaborate on the different processing and reinforcement characteristics of each filler.

## 2. Experimental

### 2.1. Materials

Nitrile butadiene rubber was supplied by Clwyd Compounders (Nipol 1052J with an acrylonitrile content of 33.5%, Wrexham, UK). The graphene nanoplatelets (GNPs) were purchased from XG Sciences, Inc., Lansing, MI, USA, and according to the supplier they exhibited nominal lateral diameters of 5 and 15 μm (M5 and M15) and average thickness in the range of 6–8 nm. Carbon black (grade N330) was obtained from Cabot (CB STD, Vale of Glamorgan, UK) and displayed a density of 1.8 g/cm^3^. All other materials were purchased from Sigma-Aldrich and used as received unless otherwise quoted.

The rubber compounds were prepared in an open two-roll mill at room temperature. The milling process was performed in the following order: firstly, the rubber was banded, then masticated, then cure activators and fillers were added, then curatives were added and finally the material was homogenised for some time before being sheeted out. The sheets were then pressed on an oil heated two-plated press at 165 °C for 7 min, followed by post-curing in an oven at 150 °C for 1 h. The mould dimensions were 150 mm × 150 mm × 2 mm. Vulcanizing conditions (temperature and time) were previously determined using a TA rheometer with a parallel plate geometry (TA Instruments, New Castle, DE, USA). The recipes of the produced compounds are listed in Table 1.

### 2.2. Characterization of the FLAKES and the Compounds

Rheology was performed using a TA instruments DHR according to ISO 3417 with a temperature of 165 °C. Test pieces were discs of 25.4 mm diameter and 2.5 mm thickness. The microstructure of the compounds was examined using scanning electron microscopy (SEM, Tescan, Brno, Czech Republic). The cryo-fractured samples were coated using an Au-Pd alloy in order to provide satisfactory conductivity to the samples. The images were acquired using a high-resolution TESCAN MIRA3 Scanning Electron Microscope, operated at 6 kV. AFM was performed to characterise the aspect ratio of the received GNPs. For this purpose, the GNPs were dispersed onto a silicon wafer by spray coating. AFM was performed using a JPK nanowizard 4 (Bruker Nano, Berlin, Germany) in tapping mode with a tip that had a nominal resonance frequency of 300 kHz and a nominal force constant of 40 N/m. Stress–strain curves were obtained using dumbbell-shaped specimens in an Instron 4301 machine (Instron, High Wycombe, UK), under a tensile rate of 500 mm·min^−1^ with a load cell of 5 kN. At least five specimens of each sample type were tested. Tear testing was performed according to BS ISO 34. Force-extension curves were obtained using angle-type test specimens in an Intron 4301 machine under a tensile rate of 500 mm·min^−1^. At least five specimens of each sample type were tested. Hardness was measured using a Rex Durometer DD-4 Type A (Buffalo Grove, IL, USA), in combination with an operating stand, performed according to ASTM D2240. Sheets were layered up to give a requisite thickness of greater than 6 mm, and for each sample, hardness was measured in three areas and average values were obtained.

## 3. Results

### 3.1. Processing Characteristics

Rheology was used to assess the optimum cure time for the rubber compound. It is important to determine a suitable cure time for all of the materials produced. By measuring the torque against time for a predetermined temperature (165 °C in this case) at a fixed shear (1%), the cure time t_95_ for all specimens was established. Table 2 below shows the average t_95_ across three specimens of each formulation. The shortest t_95_ was approximately 4 min, for NBR with 15 phr GNP or 30 phr N330. The longest t_95_ was just over 6 min for the unfilled NBR. Both carbon black and GNPs showed a reduction in t_95_ with increasing filler loading. The presence of stearic acid also appears to reduce the t_95_, as evidenced by the values for unfilled NBR.

**Figure 1:**
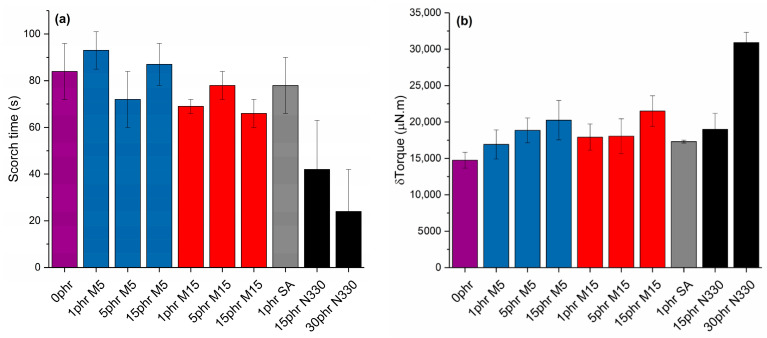
(**a**) Scorch time and (**b**) δTorque values for neat NBR and all nanocomposites under study.

The scorch time, which is the time taken until the rapid rise in torque, was found to be longer for the graphene-filled samples than for the carbon black filled samples, as shown in Figure 1a. This is a desirable feature when fabricating elastomers as it provides enough time for the rubber to fill the mould properly before curing begins and allows better control of the overall compounding process. The graphene flakes can act as a physical barrier to the sulfur accelerator reaction and the zinc–accelerator complex formation by either the adsorption of the accelerator onto the GNP flakes, resulting in reduced overall cure times [25], or due to the high surface area of the GNPs [26].

The difference between the maximum torque (MH) and the minimum torque (ML) can be considered an indication of the crosslink density as the torque measured is related to the shear modulus (and subsequently to the crosslink density). As seen from Figure 1b, with increasing loading of GNPs, the δTorque rises very slightly but is generally similar between loadings. Two competitive mechanisms are affecting the δTorque values with increasing filler content. On the one hand, GNPs reduce the friction between the hot platens of the rheometer, reducing the overall torque. On the other hand, the increase of the GNPs content can lead to higher stiffness, increasing the torque values. As a general conclusion, the introduction of GNPs with increasing filler content and diameter does not seem to impose great difficulties during processing in terms of torque, which is similar to that for the samples filled with 15 phr CB.

### 3.2. Microstructure of the NBR Nanocomposites

**Figure 2:**
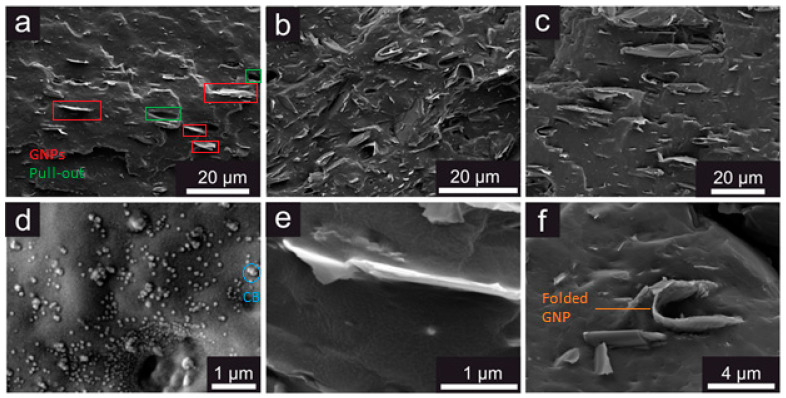
SEM images of cryo-fractured cross-sections of some of the samples under study: low magnification images of (**a**) NBR-M5 5 phr, (**b**) NBR-M5 15 phr, (**c**) NBR-M15 15 phr, high magnification image of (**d**) NBR-N330 15 phr, (**e**) a 3 μm flake within the composite showing good interface between the matrix and the filler, and (**f**) a looped/folded GNP flake within the composite.

Scanning electron microscopy (SEM) was employed to investigate the cross-sectional surfaces of cryo-fractured dumbbell samples. The results (Figure 2a–c) showed that the distribution of both M5 and M15 GNPs was quite homogeneous within the NBR matrix, while the compounding procedure attributed a certain degree of orientation to the GNPs. On the other hand, carbon black unavoidably formed some aggregates (Figure 2d), due to the strong filler-filler interactions. In terms of the interface between the filler and the matrix, the results showed that the smaller flakes displayed a better interface (Figure 2e), as a result of the ability of the rubber to wet the small nanoplatelets more effectively, compared to their larger counterparts. Moreover, another problem associated with the use of larger flakes in such materials is the tendency of the flakes to fold/bend or form looped morphologies (Figure 2b,f), which was also observed by Mondal and Khastgir [27]. This reduces the effective aspect ratio of the nanoplatelets and has a significant impact on the ultimate properties of the nanocomposites [28,29].

### 3.3. Characterisation of the Nanoplatelets

**Figure 3:**
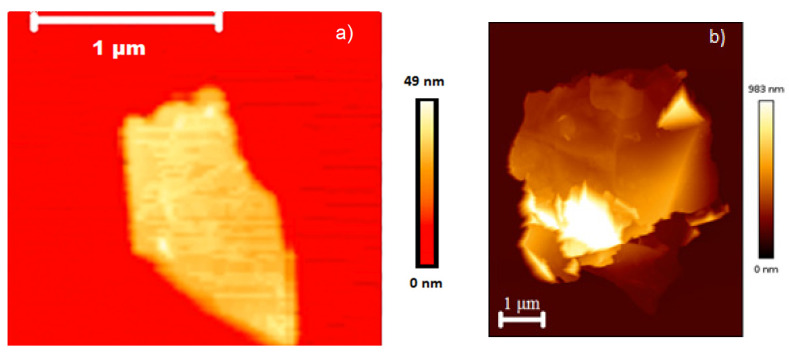
AFM images of typical M15 flakes: (**a**) a flake with an aspect ratio of 61 and (**b**) an agglomerated flake with an aspect ratio of 20.

**Figure 4:**
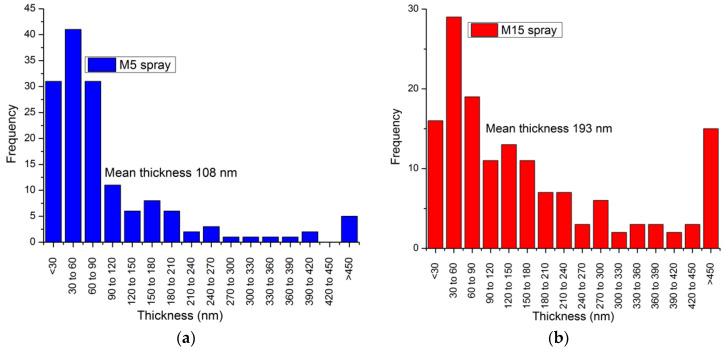
Histograms of flake thickness for (**a**) M5 and (**b**) M15 powders, as measured by AFM.

**Figure 5:**
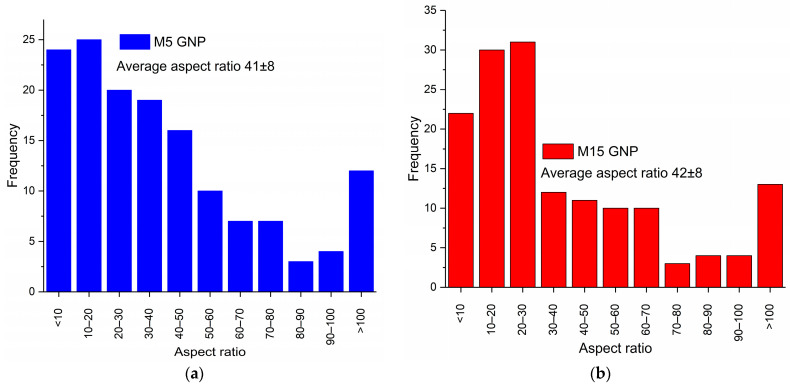
Histograms of flake aspect ratio for (**a**) M5 and (**b**) M15 powders, as measured by AFM.

Atomic force microscopy (AFM) was used to characterise the thickness and lateral dimensions of the GNPs comprehensively in order to estimate their average aspect ratio. Two different GNP flake sizes (M5 and M15) were selected so that the difference in aspect ratios could be directly measured ex-situ and in-situ and then fed into the respective micromechanics equations and discussion. The largest flakes observed for each material (M5 and M15) had a lateral size similar to the manufacturer’s quoted value of 5 μm and 15 μm, respectively, however their mean diameter was found to be significantly lower due to a high proportion of small flakes, such as that shown in Figure 3a. The mean thickness for M5 flakes was found to be 108 nm and for M15 was 193 nm, as shown by Figure 4. The histogram of aspect ratio for 150 flakes from each powder measured provides mean aspect ratio values of 41 and 42 for M5 and M15, respectively, as shown by Figure 5. Although both flakes showed a thickness in the range of 30–60 nm, similar to that reported by the manufacturer, and the largest flakes were similar in size to that reported by the manufacturer, the mean aspect ratio was far lower than that expected for the GNPs. This may be a consequence of agglomeration within the stored powder as well as agglomeration when coating onto a surface, leading to thicker-than-expected flakes such as that shown in Figure 3b. These values may be considered representative of the GNPs within the dry-mixed elastomer matrix since the obtained AFM values of diameter and thickness were similar to those observed within the NBR matrix by SEM.

### 3.4. Mechanical Properties

**Figure 6:**
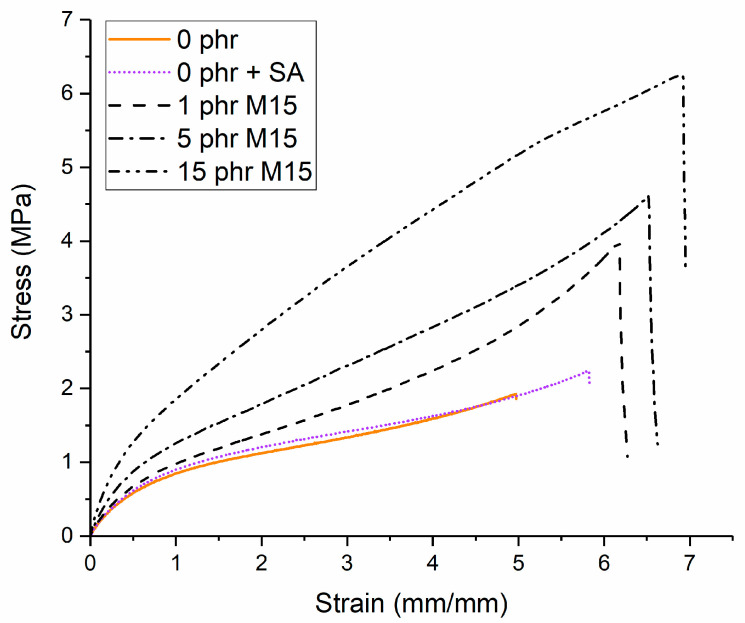
Representative stress-strain curves for Neat-NBR (with and without stearic acid) and M15-NBR (loadings 1–15 phr).

**Figure 7:**
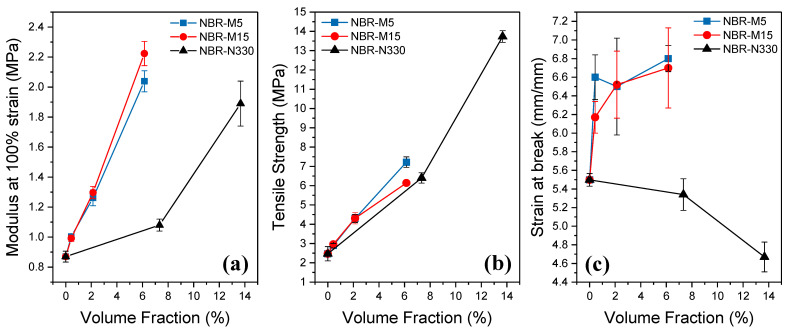
Mechanical properties of the prepared nanocomposites: (**a**) modulus at 100% strain, (**b**) tensile strength and (**c**) strain at break versus the filler volume fraction. The lines are a guide to the eye.

The mechanical properties of neat NBR and the nanocomposites were evaluated by tensile testing, and the results are presented in Figure 6 and Figure 7a–c.

Stearic acid was incorporated into the CB-NBR formulations as a processing aid. For high loadings of CB, it is often necessary to incorporate stearic acid; however, it was found to be both unnecessary and detrimental for the preparation of GNP filled samples. To demonstrate that this had little effect on the mechanical properties, Neat-NBR and NBR containing 1 phr of stearic acid are contrasted in the stress-strain curves of Figure 6. Although the strain at break appears slightly higher for the neat NBR with the inclusion of stearic acid, this was within error for the averaged set of stress-strain curves.

Figure 6 also shows the stress-strain curves for different loadings of M15-NBR, compared with Neat-NBR. In all cases, the addition of GNPs results in higher modulus and ultimate tensile strength than for neat-NBR.

Graphene nanoplatelets act as efficient reinforcing agents within the nanocomposites since the stiffness, strength and elongation of the nanocomposites all increase with increasing filler content. Regarding the modulus at 100% strain (Figure 7a), there is a steep increase with the introduction of GNPs as a result of the ability of the nanoplatelets to transfer the stress from the matrix, even though it has been shown that the low shear modulus of soft materials does not enable a highly efficient stress transfer from the matrix to the filler [10,30]. Moreover, graphene nanoplatelets may act as physical crosslinking points, a fact that can enhance the modulus even further [31]. Very small differences can be seen between the two grades of GNPs since as we saw earlier, the aspect ratio of these samples is not too different. On the other hand, carbon black does not improve the stiffness of the NBR as effectively. At similar filler contents (15 phr), the stiffness of the NBR-N330 (1.05 MPa) is half of the NBR sample reinforced with GNPs (~2.1 MPa), while even for the highest CB content (30 phr—13.5 vol%), the modulus of the sample of 1.9 MPa is still lower than the ones reinforced with GNPs.

The results of the tensile strength versus the volume fraction of the fillers presented in Figure 7b show that there are no significant differences between the two fillers and carbon black in terms of the strength of the NBR. At the same filler content (15 phr—6–7 vol%), the samples filled with M5 nanoplatelets present the highest strength values, most probably as a result of their better dispersion [32]. Finally, the ultimate strain at break of the samples presents significant differences between the two types of fillers (Figure 7c). For the case of GNPs, the strain at break increases with increasing volume fraction, contrary to what is observed for the majority of nanocomposites, as a result of the capability of the GNPs to impede crack propagation and distribute stress homogeneously for the specific set of samples. The absence of chemical crosslinks aids in maintaining the elongation at break of the GNP reinforced NBR. This phenomenon of increasing elongation at break for GNP-NBR compared with neat NBR and CB-NBR has been reported previously, especially where good alignment of the GNPs was observed [17,27,33]. It was not observed for certain solution mixing techniques [22,23,24] and so may be process-related, owing to the orientation of the GNPs.

Additionally, a number of studies also state that carbon black can provide chemical crosslinks with the rubber [34,35,36,37,38], which would be expected to reduce the elongation at break. Furthermore, the agglomerates of carbon black that are formed in the NBR-CB samples can be also considered responsible for the reduction in elongation because they acts as points of failure.

### 3.5. Tear Properties

**Figure 8:**
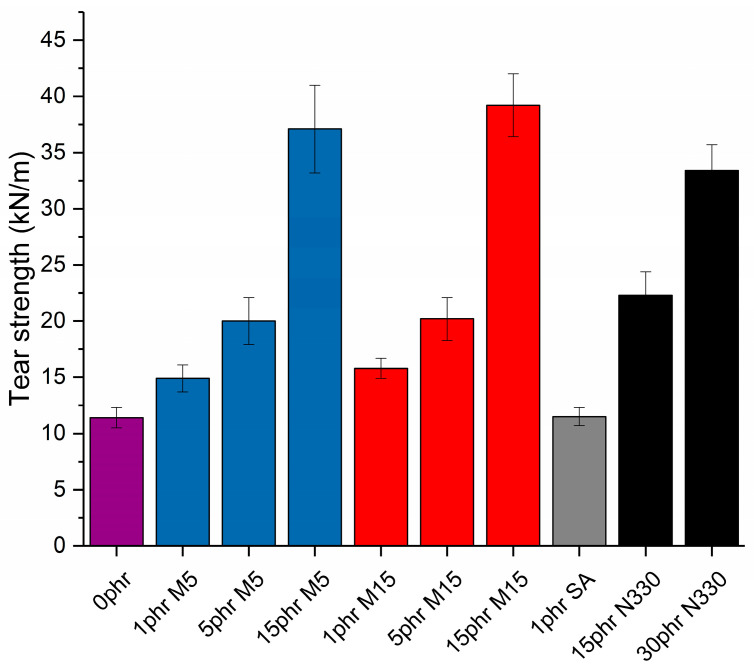
Tear strength values for neat NBR and all nanocomposites under study.

The tear strength, a measure of the resistance of the nanocomposites to the applied tear force, was evaluated for all samples. From the results presented in Figure 8, it can be seen that the tear strength of the GNP-reinforced nanocomposites increases compared to neat NBR with increasing filler content: at 15 phr of either M5 or M15 GNPs, the strength was almost four times higher. In comparison, the strength of the samples filled with carbon black at both 15 and 30 phr filler content was always lower than that of the samples filled with 15 phr GNPs, indicating once again the efficiency of GNPs to support the stress that is transferred from the elastomer to the flakes. This can also be correlated with the compatibility between the GNPs and the elastomers and the subsequent increase in stiffness of the nanocomposites from the introduction of the nanoplatelets. The tear energy of a composite can be a combination of different mechanisms such as crack deflection, debonding, pull-out and void growth [39]. As shown earlier, the GNP-reinforced samples display higher elongation at break compared to neat NBR and the NBR-CB samples since the flakes reduce the crack propagation in the nanocomposites due to their inherent properties and geometrical characteristics; large platelets are expected to retard crack growth more greatly and therefore give a greater tear strength. This is in agreement with the work of Chong et al. who found crack deflection to be the main fracture energy contribution for their GNP-reinforced epoxy [40]. Recently, Liu et al. evaluated the tearing of graphene-reinforced elastomers under pure shear tests, demonstrating significant debonding at the interface between the nanoplatelets and the elastomeric matrix [36].

### 3.6. Hardness

**Figure 9:**
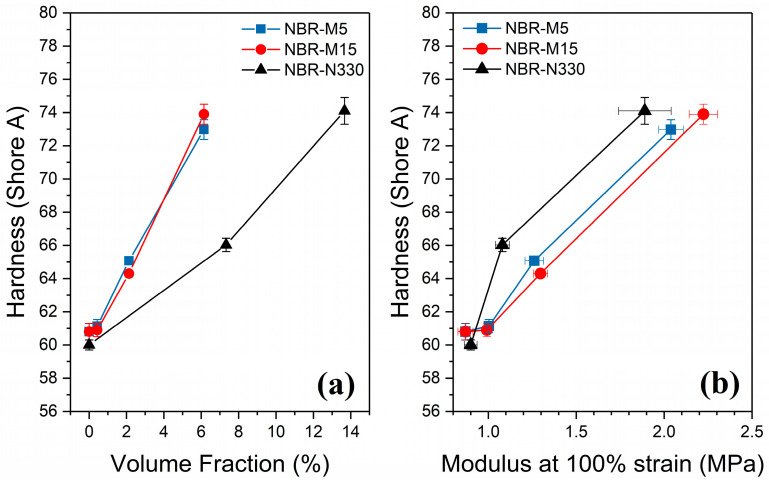
(**a**) Hardness comparison between the samples under study against volume fraction; (**b**) cross-plot of hardness versus modulus.

The hardness of the elastomer, a measure of the nanocomposite surface’s resistance to an instantaneous indentation, was evaluated for all samples. For unfilled rubber, the hardness is believed to be strongly related to the crosslink density [41]. The hardness for the NBR-GNP composites is greater than the corresponding NBR-CB, as shown by Figure 9a. This may be attributed to the significantly higher aspect ratio of the GNPs, which allows for a more effective stress transfer.

As shown by Figure 9b, the modulus increases more rapidly than the hardness for the NBR-GNP compared with NBR-CB. If two samples of equivalent hardness were produced, then the GNP-reinforced elastomer would have greater stiffness, and the same is found for tear strength. Additionally, at low loadings (1 phr GNP), the sample sees a rapid increase in modulus but only a small increase in hardness. This may suggest that adding a small amount of GNP to an existing NBR-CB compound could give a significant increase in the stiffness with little change in hardness. The hardness values may be less affected by the aspect ratio of the reinforcing filler than the modulus, although this could be orientation dependent. The GNPs are orientated perpendicular to the direction of impact from the durometer yet in the plane of the tensile direction.

## 4. Discussion

### Composite Micromechanics

The mechanical properties of the NBR composites reinforced by GNPs and CB were also analysed using micromechanical theories. According to the well-accepted rule-of-mixtures, the modulus of the composites is given by [42]:

(1) $$E_{c}{=E}_{f}V_{f}{+E}_{m}V_{m}$$

where *E*_f_ and *E*_m_ are the modulus of the filler and the matrix, while *V*_f_ and *V*_m_ are the volume fraction of the filler and the matrix, respectively. Based on Equation (1), linear fittings were carried out for the mechanical properties (normalised modulus against volume fraction of the filler) of the NBR/GNP composites. The slopes of the fitted lines indicate the individual filler modulus of the two different types of GNPs. The calculated filler modulus (*E*_f_) based on the rule-of-mixtures for M5-GNPs is 19 MPa and the corresponding value for M15-GNPs is 22 MPa. These two values are well below the value of ~1 TPa reported for the Young’s modulus of monolayer graphene [5,43]. This fact clearly indicates that although the improvement of the mechanical properties of elastomer materials reinforced by graphene, in terms of percentage increase, can be considered significant, the actual reinforcement procedure that takes place through stress transfer from the soft elastomer to the stiff nanoplatelets is rather inefficient. As we have pointed out recently, the graphene filler modulus scales with the matrix modulus and the exceptional mechanical properties of graphene (and graphene-related materials) cannot be utilised in composites where the shear modulus of the matrix is very low, such as elastomers [10].

In the specific case of GNP-reinforced elastomers, where the modulus of the matrix is significantly lower than that of the reinforcement, the filler modulus (*E*_f_) is dependent upon the orientation factor (*η*_o_), aspect ratio (*s*) and volume fraction of the filler, while it is independent of the modulus of the nanoplatelets [10,30]. The filler modulus (*E*_f_) is given by:

(2) $$E_{f}{=\eta }_{o}\frac{s^{2}}{12}\frac{t}{T}\frac{1}{\left(1+\nu \right)}E_{m}$$

where *s* is the aspect ratio of the GNPs; *ν* is the Poisson’s ratio of the elastomer; *t* is the thickness of the flakes; and *T* is the thickness of the matrix surrounding the flakes, which can be affected by the flakes during deformation. By combining Equations (1) and (2), the normalised modulus (*E*_c_/*E*_m_) of the composites is given by:

(3) $$E_{c}{/E}_{m}{=1+(\eta }_{o}\frac{s^{2}}{12}\frac{t}{T}\frac{1}{\left(1+\nu \right)}{\;-\;1)V}_{f}$$

With regard to NBR reinforced by GNPs, where the matrix is very flexible even among elastomers and the shear modulus is very low, it is reasonable to assume that each individual flake can only affect a very thin layer (*T* is very small) of the surrounding matrix. Therefore, *t*/*T* may be considered to be constant and remains unaffected when the filler loading increases up to 15 phr (*V*_f_ ≈ 0.06). When the filler loading is high enough and the mechanical percolation threshold volume fraction is reached, making *t*/*T* ≈ *V*_f_, then the normalised modulus is given by [10,30]:

(4) $$E_{c}{/E}_{m}{=1\;-\;V}_{f}{+\eta }_{o}\frac{s^{2}}{12}\frac{1}{\left(1+\nu \right)}V_{f}^{2}$$

Equation (4) shows a quadratic relationship between the normalised modulus of the composites (*E*_c_/*E*_m_) against the volume fraction of the filler (*V*_f_), and it was recently employed to analyse a thermoplastic elastomer reinforced by GNPs [30]. This quadratic relationship can be interpreted on the basis of the accelerated stiffening phenomenon, where after a certain volume fraction, the increase of the normalised modulus of the composites is parabolic. However, this equation cannot be applied in the case of NBR reinforced by GNP, possibly due to the more flexible nature of the polymer, which eventually leads to a high mechanical percolation threshold volume fraction (higher than 15 phr). Therefore, the data were analysed using only Equation (3).

**Figure 10:**
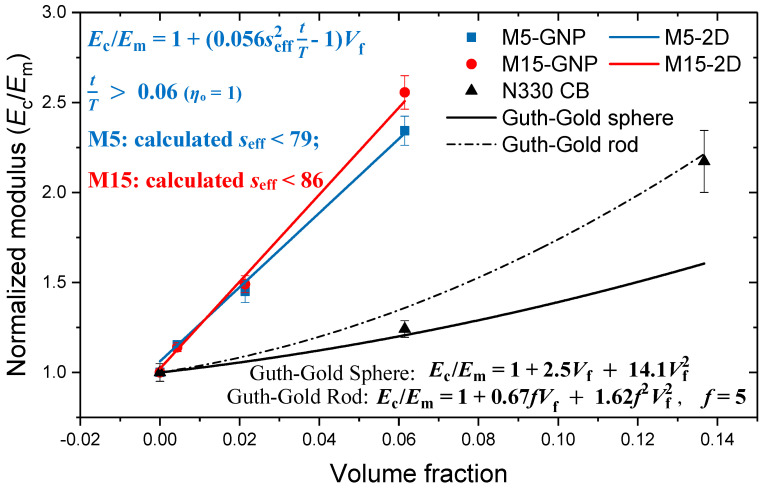
Fitting of the experimental data (solid symbols) with the micromechanical theories developed for the reinforcement of polymers from 2D materials by Young et al. [10] for the case of oriented nanoplatelets and from spheres/rods by Guth and Gold [45].

Assuming the Poisson’s ratio of NBR is 0.5, the orientation factor (*η*_o_) being 1 for perfect orientation of the GNP flakes (Figure 10) and 0.53 for random orientation of the flakes [44] (Figure S1-Supplementary Information), Equation (3) can be rewritten as:

(5) $$E_{c}{/E}_{m}=1+(0.056s_{\mathrm{eff}}^{2}\frac{t}{T}{\;-\;1)V}_{f}\;(\eta_{o}=1)\;\mathrm{perfect}\;\mathrm{orientation}\;\mathrm{of}\;\mathrm{the}\;\mathrm{filler}$$

(6) $$E_{c}{/E}_{m}=1+(0.029s_{\mathrm{eff}}^{2}\frac{t}{T}{\;-\;1)V}_{f}\;(\eta_{o}=0.53)\;\mathrm{random}\;\mathrm{orientation}\;\mathrm{of}\;\mathrm{the}\;\mathrm{filler}$$

where *s* is the effective aspect ratio of the flakes, contributing to the enhancement of the modulus. Linear fittings of the normalised modulus against volume fraction of the filler were carried out based on Equations (5) and (6). The slopes of the fitted lines in Figure 10 and Figure S1 give fixed values of $(0.056s_{\mathrm{eff}}^{2}\frac{t}{T}-1)$ and $(0.029s_{\mathrm{eff}}^{2}\frac{t}{T}-1)$ for the assumption of perfect orientation and random orientation of the flakes, respectively. Then, the effective aspect ratio (*s*_eff_) can be calculated using the *t*/*T* value of 0.06, which is the highest loading of the samples we prepared, and, ultimately, the *s*_eff_ values are in the order of 80 for perfect orientation of the filler, while the corresponding value for the random orientation of the filler is around 120. The fitted results show good consistency with our previous work [30].

The mechanical properties of the carbon-black-reinforced composites were also analysed using the classic Guth–Gold theory, which is based on hydrodynamics [45]. It proposes that when the loading of the carbon black in the rubber composites is low and the dispersion is homogeneous, the reinforcement depends on the reinforcement efficiency of the individual spheres, and the normalised modulus is given by:

(7) $$E_{c}{/E}_{m}=1+2.5V_{f}+14.1V_{f}^{2}$$

where *V*_f_ is the volume fraction of the carbon black. Is should be noted that this equation also implies that the modulus of the nanocomposite depends only upon the modulus of the rubber and not upon the modulus of the filler. When the loading of the carbon black is high enough, the carbon black spheres form agglomerates of rod-like shapes, giving rise to additional contribution to the stiffness of the materials. As it was mentioned earlier, this phenomenon is termed accelerated stiffening, resulting from the mutual interaction of the spheres, and then the normalised modulus of the composites is given by:

(8) $$E_{c}{=E}_{m}(1+0.67{fV}_{f}+1.62f^{2}V_{f}^{2})$$

where *f* is the shape factor (*f* = length/breadth of the rod). It can be seen that Equation (7) fits the modulus of 15 phr carbon-black-reinforced NBR and Equation (8) fits the modulus of 30 phr carbon-black-reinforced NBR when we set the shape factor *f* as 5 (Figure 10). It was previously found that the modification of the Guth–Gold equation with the shape factor (Equation (8)) works accurately, when *f* is around 6 [46], a value very close to the one we obtained for the NBR-CB nanocomposites. The shape factor obtained may be related to the grade of carbon black used. This can be compared with the GNPs where the form of the equation is similar, but the shape factor is now replaced by the effective aspect ratio, which for GNPs M5 and M15 is estimated to be 79 and 86, respectively, as shown in Figure 10.

Overall, it can be concluded that due to the geometrical characteristics of the nanoplatelets and their inherent mechanical properties, along with the homogeneous dispersion and the stronger interfaces, a higher degree of enhancement in the stiffness of the materials was achieved for GNP-reinforced NBR nanocomposites, compared to the carbon black ones.

It is worth noting that the calculated aspect ratio of ~80 is within the same order of magnitude as that measured by AFM. The comprehensive characterization by AFM validates the fitting, and together the results demonstrate that the agglomeration of nanoplatelets significantly affects the elastomer nanocomposite modulus. Whilst it has previously been widely reported that high loadings of GNPs a drop-off in mechanical properties take place due to agglomeration, the loading at which this occurs varies [24,25,47,48]. The reason for this is now clear. The preparation of the GNPs in terms of their diameter and thickness, as well as their orientation and dispersion within the matrix, affect their effective aspect ratio. Depending on how the GNPs agglomerate during dispersion, the optimum loading will vary. This can be demonstrated by taking values of loading versus modulus from the literature and estimating the expected aspect ratio using Equation (5), as shown in Table 3.

The GNPs used by Mondal and Varghese were from the same supplier as used in this paper; hence, the estimated aspect ratio is similar [17,27]. The results from Thomas and Frasca indicate that the use of solution blending prior to milling helps to prevent agglomeration of GNPs (although mainly at low loadings) [48,49]. The best mechanical reinforcement is expected for flakes with the highest aspect ratio; however, preventing agglomeration of GNPs, especially at high loadings, during processing such as in a two roll mill, remains a significant challenge.

## 5. Conclusions

The differences in the reinforcement characteristics of graphene nanoplatelets and carbon black within a nitrile butadiene rubber matrix have been evaluated in detail. From rheological measurements, we concluded that the curing times were reduced and the scorch time was approximately doubled in the case of NBR-GNP nanocomposites compared with NBR-CB but not at the expense of the torque because both fillers displayed similar torque values for the same filler loadings. The dispersion of GNPs was homogeneous for both larger and smaller GNPs, while the interface (as seen by SEM) between the smaller flakes and the matrix was better than the large flakes since the rubber can flow efficiently around the nanoplatelets. Agglomerated flakes were identified both in the GNP powders, as measured by AFM, and within the elastomer matrix. Another problem associated with the use of large flakes for the reinforcement of rubbers is their bending or folding during processing, which eventually reduces their aspect ratio. Overall, the aspect ratio of the GNPs was found to be in the range 40–90, which is lower than anticipated from the manufacturer’s values. It has been demonstrated that a higher aspect ratio GNP will mechanically reinforce the matrix more efficiently by comparing GNP M5 with M15. We anticipate that this should lead researchers towards the optimization of the GNP fabrication methods to achieve nanoplatelets with larger lateral sizes and smaller thicknesses (thus achieving higher aspect ratios).

In terms of mechanical properties, the NBR-GNP composites were stiffer and more flexible, compared to the NBR-CB samples. At 15 phr, GNP-NBR showed double the Young’s modulus of CB-NBR (2.1 vs. 1.05 MPa). Moreover, the tear strength of the GNP-reinforced composites (38 kN/m at 15 phr) was higher than the NBR-CB (33 kN/m at 30 phr), as a result of the ability of the GNPs to obstruct crack propagation. Additionally, the introduction of a small amount of GNPs saw significant increases in modulus and tear strength with a comparatively insignificant change in hardness. It was demonstrated by micromechanical modelling how the GNP-reinforced NBR achieved these mechanical properties. Overall, we have shown that the introduction of GNPs within NBR offers significant advantages over using carbon black because the majority of the physicochemical properties evaluated here were improved, while the use of GNPs enabled easier processing of the nanocomposites. GNP-NBR may be beneficial for applications where reduced hardness and increased elongation at break are desired compared with CB-NBR. Specifically, for the equivalent tear resistance, the GNP reinforced NBR is softer, which could help reduce damage during installation for the oil and gas industry.

## Figures and Tables

**Table 1:** Recipes of the rubber compounds (indicated in phr).

Compound	NBR (phr)	ZnO (phr)	S (phr)	CBS (phr)	TMTD (phr)	SA (phr)	xGnP (phr)	CB (phr)
NBR-unfilled	100	3	2	0.5	0.25	0	0	0
NBR-SA-unfilled	100	3	2	0.5	0.25	1	0	0
NBR-M5-1:15	100	3	2	0.5	0.25	0	M5: 1,5,15	0
NBR-M15-1:15	100	3	2	0.5	0.25	0	M15: 1,5,15	0
NBR-SA-N330	100	3	2	0.5	0.25	1	0	N330: 15,30

**Table 2:** Torque values and cure times (t_95_) for all compounds under study. The deviation in the quoted values is in the order of 10%.

Compound	Maximum Torque (μN·m)	Minimum Torque (μN·m)	δTorque (μN·m)	t_95_ (min)
0 phr	16,100	1300	14,800	6.1
1 phr SA	19,000	1700	17,300	4.2
1 phr M5	18,500	1500	16,900	5.9
1 phr M15	19,600	1600	17,900	5.6
5 phr M5	20,700	1800	18,900	4.5
5 phr M15	19,700	1700	18,000	4.8
15 phr M5	22,500	2300	20,200	4.1
15 phr M15	24,100	2600	21,500	4.1
15 phr N330	21,100	2100	19,000	5.4
30 phr N330	48,700	17,800	30,900	4.3

**Table 3:** Fitting of the literature results with Equation (5) relating to the reinforcement of NBR by GNPs.

Authors	GNPs	Processing Method	*V*_f_ Filler (%)	Modulus_initial_ (MPa)	Modulus_final_ (MPa)	Estimated Aspect Ratio
Varghese et al. [17]	XG Sciences	2-roll mill	0.41	12.75	14.27	94
			1.21	12.75	16.9	90
			2.00	12.75	19.7	90
Mondal et al. [27]	XG Sciences	Solution/2-roll mill	0.40	0.84	1.18	175
			1.98	0.84	1.48	107
			5.70	0.84	2.87	112
			9.16	0.84	4.04	111
Thomas et al. [48]	Prepared FLG	2-roll mill	1.98	2.42	1.7	130
			3.88	2.42	2.4	124
			5.70	2.42	2.6	108
			7.46	2.42	1.9	74
Frasca et al. [49]	Graph. K. MLG	Solution/2-roll mill	1.22	1.68	4.03	185

## Data Availability

The data presented in this study are available on request from the corresponding author.

## Supplementary Materials

The following supporting information can be downloaded at: https://www.mdpi.com/article/10.3390/polym14061204/s1, Figure S1: Micromechanical modelling of the reinforcement of polymers by 2D materials using random orientation.
