# Supplementary material for: Graphene Nanoplatelets as a Replacement for Carbon Black in Rubber Compounds
Source: Polymers (Basel). 2022 Mar 17;14(6):1204. doi: 10.3390/polym14061204 (PMC8949821; doi:10.3390/polym14061204)
Supplement: Supplementary file 1 [file polymers-14-01204-s001.zip › polymers-1611723-supplementary.pdf]

polymers

MDPI

Supplementary Information

Graphene Nanoplatelets as a Replacement for Carbon Black in Rubber Compounds

James R. Innes [1,2], Robert J. Young [2] and Dimitrios G. Papageorgiou [2,3,*]

[1] Polymer IRC, Faculty of Engineering and Informatics, University of Bradford, Richmond Road, Bradford BD71DP, UK; j.innes1@bradford.ac.uk
[2] National Graphene Institute, Henry Royce Institute and Department of Materials, University of Manchester, Oxford Road, Manchester M13 9PL, UK; robert.young@manchester.ac.uk
[3] School of Engineering and Materials Science, Queen Mary University of London, London E1 4NS, UK
* Correspondence: d.papageorgiou@qmul.ac.uk

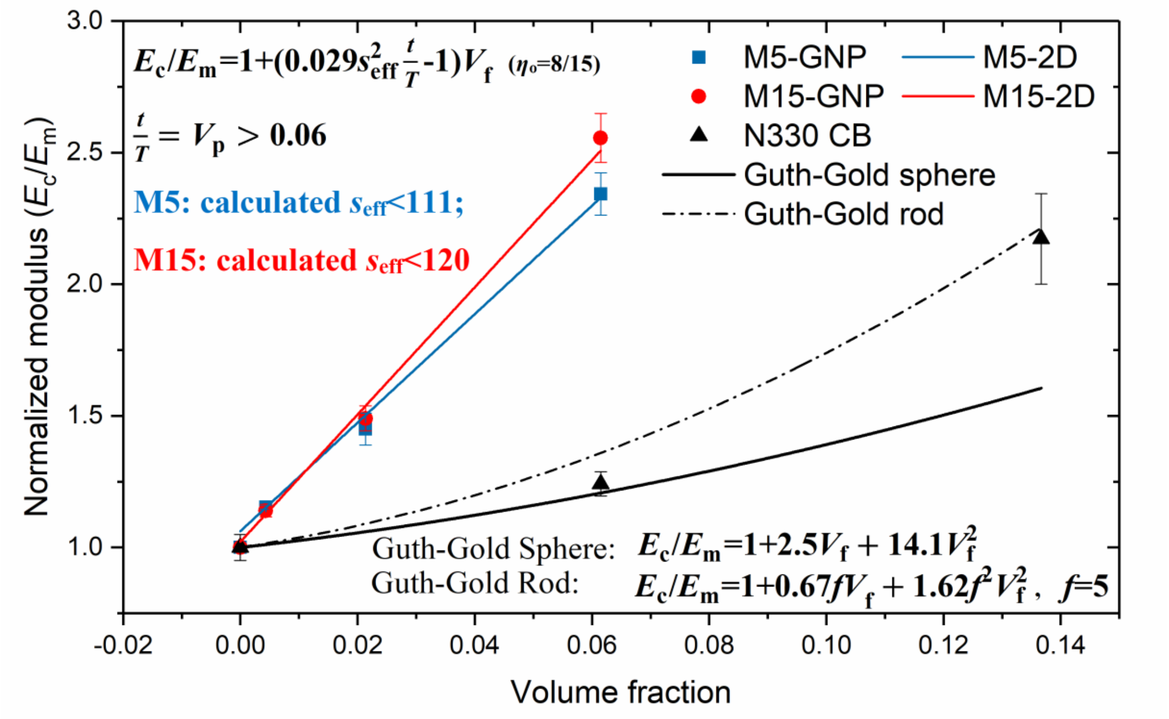

Figure S1. Fitting of the experimental data (solid symbols) with the micromechanical theories developed for the reinforcement of polymers from 2D materials by Young et al. [10] for the case of randomly oriented nanoplatelets and from spheres/rods by Guth & Gold [37].

The slopes of the fitted lines in Figure S1 uses $(0.029s_{eff}^2\frac{t}{T}\text{-}1)$ for the assumption of random orientation for the reinforcing GNPs. This can be contrasted with the perfect orientation assumed in Figure 10. Perfect orientation was considered more appropriate for the paper since the SEM results showed alignment of the GNPs within the NBR matrix. The effective aspect ratio (s_{eff}) was calculated using the t/T value of 0.06, which is the highest loading of the samples we prepared, and, ultimately, the s_{eff} values are in the order of 110-120 for random orientation, which is higher than the calculated values for perfect orientation (~80). Therefore, it is recommended to measure both orientation/alignment and aspect ratio of GNPs within the elastomer matrix when modelling the reinforcement obtained. The fitted results show good consistency with our previous work [30].
